# The wheat pathogen *Zymoseptoria tritici* senses and responds to different wavelengths of light

**DOI:** 10.1186/s12864-020-06899-y

**Published:** 2020-07-25

**Authors:** Cassandra B. McCorison, Stephen B. Goodwin

**Affiliations:** 1 Department of Botany and Plant Pathology, Purdue University, 915 West State Street, West Lafayette, IN 47907-2054 USA; 2 USDA-Agricultural Research Service, Crop Production and Pest Control Research Unit, Department of Botany and Plant Pathology, Purdue University, 915 West State Street, West Lafayette, IN 47907-2054 USA

**Keywords:** Gene expression, Light, *Mycosphaerella graminicola*, Photobiology, RNAseq, *Zymoseptoria tritici*

## Abstract

**Background:**

The ascomycete fungus *Zymoseptoria tritici* (synonyms: *Mycosphaerella graminicola, Septoria tritici*) is a major pathogen of wheat that causes the economically important foliar disease Septoria tritici blotch. Despite its importance as a pathogen, little is known about the reaction of this fungus to light. To test for light responses, cultures of *Z. tritici* were grown in vitro for 16-h days under white, blue or red light, and their transcriptomes were compared with each other and to those obtained from control cultures grown in darkness.

**Results:**

There were major differences in gene expression with over 3400 genes upregulated in one or more of the light conditions compared to dark, and from 1909 to 2573 genes specifically upregulated in the dark compared to the individual light treatments. Differences between light treatments were lower, ranging from only 79 differentially expressed genes in the red versus blue comparison to 585 between white light and red. Many of the differentially expressed genes had no functional annotations. For those that did, analysis of the Gene Ontology (GO) terms showed that those related to metabolism were enriched in all three light treatments, while those related to growth and communication were more prevalent in the dark. Interestingly, genes for effectors that have been shown previously to be involved in pathogenicity also were upregulated in one or more of the light treatments, suggesting a possible role of light for infection.

**Conclusions:**

This analysis shows that *Z. tritici* can sense and respond to light with a huge effect on transcript abundance. High proportions of differentially expressed genes with no functional annotations illuminates the huge gap in our understanding of light responses in this fungus. Differential expression of genes for effectors indicates that light could be important for pathogenicity; unknown effectors may show a similar pattern of transcription. A better understanding of the effects of light on pathogenicity and other biological processes of *Z. tritici* could help to manage Septoria tritici blotch in the future.

## Background

Light is essential for many biological processes and is an important environmental cue. Fungi have multiple responses to light, which can vary from species to species. Much of the research done on light responses in ascomycetes has been with the model filamentous fungus *Neurospora crassa* [1–9]. This species has clearly delineated morphology between growth that occurs in the light versus the dark [1–6].

Multiple species in a variety of families rely on light to regulate the machinery that helps them cope with stresses. UV radiation damages DNA, and the cells respond by producing photolyase proteins to repair the damage. The expression of photolyases is often induced by light, as it is only required after exposure to UV [10–14]. Oxidative stress, which can be caused by light, relies on light-sensing genes to induce production of proteins required for cellular repair [15, 16]. Many fungi are protected from photobiological damage by pigments, such as melanin and various carotenoids, production of which can be induced by light [2, 16–21].

Light can have major effects on fungal growth and morphology. In some species of fungi, light induces asexual reproduction via the formation and germination of conidia, and represses sexual reproduction [22–27]. Yet the opposite pattern is found in other species [17, 28–30]. Growth in day:night cycles can lead to fungal colonies showing concentric circles of differing morphologies on agar plates [17, 31, 32]. Presumably this also would occur within plant hosts and may explain the spread of lesions in concentric circles seen in fungi such as many species in the genus *Alternaria*, some of which show maximum growth in culture under alternating cycles of light and dark [32].

In some fungal species, light regulates when toxins and other secondary metabolites are produced [30, 33–38]. In *Cercospora* species that produce the light-activated phytotoxin cercosporin, light induces production of the toxin, as that is when it is most effective [39, 40], and no cercosporin is produced in the dark. A similar phenomenon occurs in *Alternaria alternata* where production of the mycotoxins altertoxin and alternariol are induced exclusively or have expression increased greatly under blue light in contrast to dark [41]. Similarly, the production of aflatoxin in *Aspergillus* species can be affected by both the color and intensity of light, although the conditions under which mycotoxins are produced at the highest rates are not uniform within the genus [35, 42]. Alternatively, in *Fusarium graminearum*, light represses the production of the trichothecene mycotoxins deoxynivalenol and 15-acetyl-deoxynivalenol [43].

Curiously, the ability to sense light also is required for pathogenicity and virulence in some pathogenic fungal species. For example, in the plant pathogens *Botryis cinerea* and *Cercospora zea-maydis*, knocking out *wc1*, the light-sensing component of the proteins comprising the White Collar Complex (WCC), leads to lowered virulence or a complete lack of pathogenicity [15, 44]. In *Aspergillus flavus*, the deletion of *wc1* or *velvet* homologs drastically reduce the ability of the fungus to infiltrate corn kernels, peanuts, and cotton bolls [30, 37, 38]. However, in the rice pathogen *Magnaporthe oryzae*, light represses infection, and leads to much lower disease severity [45]. A *wc1* knockout in *M. oryzae* showed greater disease severity, as it could infect in the light as well as in the dark, compared to wild type which only infects in the dark [45].

*Zymoseptoria tritici* (synonyms: *Mycosphaerella graminicola, Septoria tritici*) is the causal agent of Septoria tritici blotch, an economically important disease of wheat. Losses due to this disease can reach up to 50% in epidemic years, and often vary between 5 and 20% depending on the environment and the cultivar of wheat; it has been estimated that up to 70% of fungicide use in Europe is to control this disease [46–48]. Spores of *Z. tritici* are splash dispersed during rainstorms, and need humid conditions for successful infection [49]. After landing on a leaf, the spores germinate, and invade the wheat plants via the stomata [50]. Initial growth appears to be biotrophic, but the fungus rapidly switches to necrotrophic growth beginning 12–14 days after penetration [51]. Controlling *Z. tritici* is becoming more difficult, as resistance to the strobilurin (quinone-outside inhibitor) fungicides has become widespread in Europe, and also has appeared in North America [48, 52]. Other effective fungicides are the demethylation inhibitors (azoles), the SDHIs (succinate dehydrogenase inhibitors), and multi-site fungicides [48]. Resistance to the azoles has already begun to spread, and the SDHI fungicides are at a medium to high risk of fungal populations developing resistance [48, 53–56]. Understanding more about how *Z. tritici* infects wheat and what conditions are necessary for it to reproduce are critical for developing better methods of disease control.

Currently, little is known about how *Z. tritici* senses and responds to light. Light does have minor effects on the growth and development of *Z. tritici*, stimulating formation of aerial hyphae, and prolonging the time growing in yeast-like form before transitioning to hyphal form [57, 58]. Light is a very important environmental cue for some fungi in the Dothideomycetes, the class that contains *Z. tritici* and many other important plant pathogens [59, 60]. For example, in the genus *Cercospora*, another genus in the same taxonomic order (Capnodiales), light represses asexual sporulation, and melanization is controlled by circadian rhythms [17, 44]. The disruption of *CRP1*, a homolog of *wc1* in *N. crassa*, eliminates the stomatal tropism that *C. zea-maydis* needs to infect maize [44]. The conidiation and growth of two species in the order Pleosporales, *Alternaria alternata* and *Exserohilum turcicum*, are also regulated by light [41, 61]. Additionally, as mentioned previously, light regulates toxin production in multiple Dothideomycetes species [33, 40, 41, 62].

The *Z. tritici* genome [63] contains genes coding for a number of homologs to photoreceptors in other fungi. These include homologs for the two White Collar Complex genes (*wc1* and *wc2*), VIVID, a blue-light-sensing cryptochrome, a photolyase plus two putative photolyase genes and a red-light-sensing phytochrome [10, 25, 64, 65]. It also has homologs of some genes that may not be involved in sensing light, but do respond to light in other species, such as *frq*, a circadian rhythm gene that codes for the frequency clock protein, and *velvet*, which responds to light and has been shown to react with a phytochrome and the white collar complex in *Aspergillus nidulans* [66–69]*.* Knocking out function of the velvet gene, *MVE1*, in *Z. tritici* had large effects on growth and development, including increased sensitivity to stresses, reduced melanin production, and blindness to light-induced aerial hyphae formation [57].

Between the evidence that closely related fungi can sense light and the presence of putative photoreceptors and photoreceptor homologs, it seems highly likely that *Z. tritici* can sense and respond to light, but this has not been tested specifically. The goal of this research was to test the hypothesis that *Z. tritici* can sense and respond to light. Secondary goals were to identify genes that are highly regulated by light for future study and to augment the annotation of the reference genome by analyzing the expression of genes induced by different wavelengths of light.

## Results

### RNA sequencing

RNA samples extracted from mycelia grown in three biological replicates of four light growth conditions, 16 h per day of white, blue (400–530 nm), or red (600–700 nm) light and continuous darkness, were sequenced and the poly A, unstranded reads were filtered for quality, mapped, and analyzed. On average, 1.35% of all sequence reads were too short after trimming and were removed from each of the samples, and an average of 98.7% of the reads mapped to the *Z. tritici* genome annotations (Table 1). The remaining 1.3% of the reads mostly appeared to be contaminants. When the read counts for each gene were plotted between the three biological replicates of each condition, the reproducibility was very high (Additional File 1A). The lowest variability appeared to occur between the three white light replicates, while the greatest was between the first and third dark replicates (Additional File 1A); however, even this was low compared to the variability among the different treatments.

**Table 1:** Summary statistics about RNA sequence reads and mapping to the reference genome for *Zymoseptoria tritici* cultures exposed to different light treatments

Treatment	Replicate	Total raw reads (millions)	Reads mapped to reference genome (millions)	Percentage of reads mapped to reference genome
White light	1	32.50	31.32	97.8
	2	45.33	41.32	92.7
	3	40.20	38.72	97.8
Blue light	1	42.21	40.72	97.9
	2	32.52	31.33	97.8
	3	55.45	53.49	97.9
Red light	1	44.38	42.86	98.0
	2	43.32	41.65	97.7
	3	36.64	35.41	98.1
Dark	1	156.64	154.20	99.2
	2	114.25	112.89	99.7
	3	152.06	150.29	99.7

Out of 13,522 total genes called in the reference genome, 12,079 (89%) had greater than 10 reads in each replicate and more than 100 reads across all twelve replicates, and were used in analysis with DESeq2. The cleaned data were distributed normally, both within individual replicates and as a whole (Additional File 1B).

### Differential gene expression in response to light

There were major differences in the numbers of differentially expressed genes (adjusted *p* value < 0.05 and an absolute log2 fold change (LFC) > 2) between the dark condition and any of the three light treatments, with all three of the light conditions versus dark having over 2000 differentially expressed genes each, while white versus red was the only other comparison to break 100 differentially expressed genes (Table 2). The white light versus red light comparison was distinct from the white light versus blue light and red light versus blue light comparisons, as well as from the light vs dark comparisons (Table 2). All six comparisons between the treatments showed the expected concavity in the volcano plots, but the magnitudes of the adjusted *p* values were much greater in the three light versus dark comparisons, with the lowest magnitude of adjusted *p* values in white light versus blue light (Additional File 1C).

**Table 2:** Differentially expressed genes between cultures of *Zymoseptoria tritici* exposed to dark or different wavelengths of light

Criterion^a^	White vs dark	Blue vs dark	Red vs dark	White vs blue	White vs red	Blue vs red
LFC 2 Up	1524	1334	1207	4	224	72
LFC 2 Down	1900	1527	1296	140	563	24
Total LFC 2	3424	2861	2503	144	787	96
% annotated^b^	51.6	50.3	48.9	36.8	39.5	45.8

^a^ Criteria were adjusted *p* value < 0.05 and an absolute log2 fold change (LFC) > 2.

^b^ % annotated indicates genes with functional annotations using GO, KEGG or EggNOG annotations

In total, 4187 unique genes were differentially expressed between all six comparisons. This is just under a third (31%) of all genes present in the *Z. tritici* genome. In the three light versus dark comparisons, 4019 unique genes were differentially expressed, which is very near the total unique genes, and still represents 30% of all genes in *Z. tritici*. In the three light versus light comparisons, 804 unique genes were differentially expressed, which is only about 6% of the genes in the *Z. tritici* genome (Additional File 2).

**Fig. 1:**
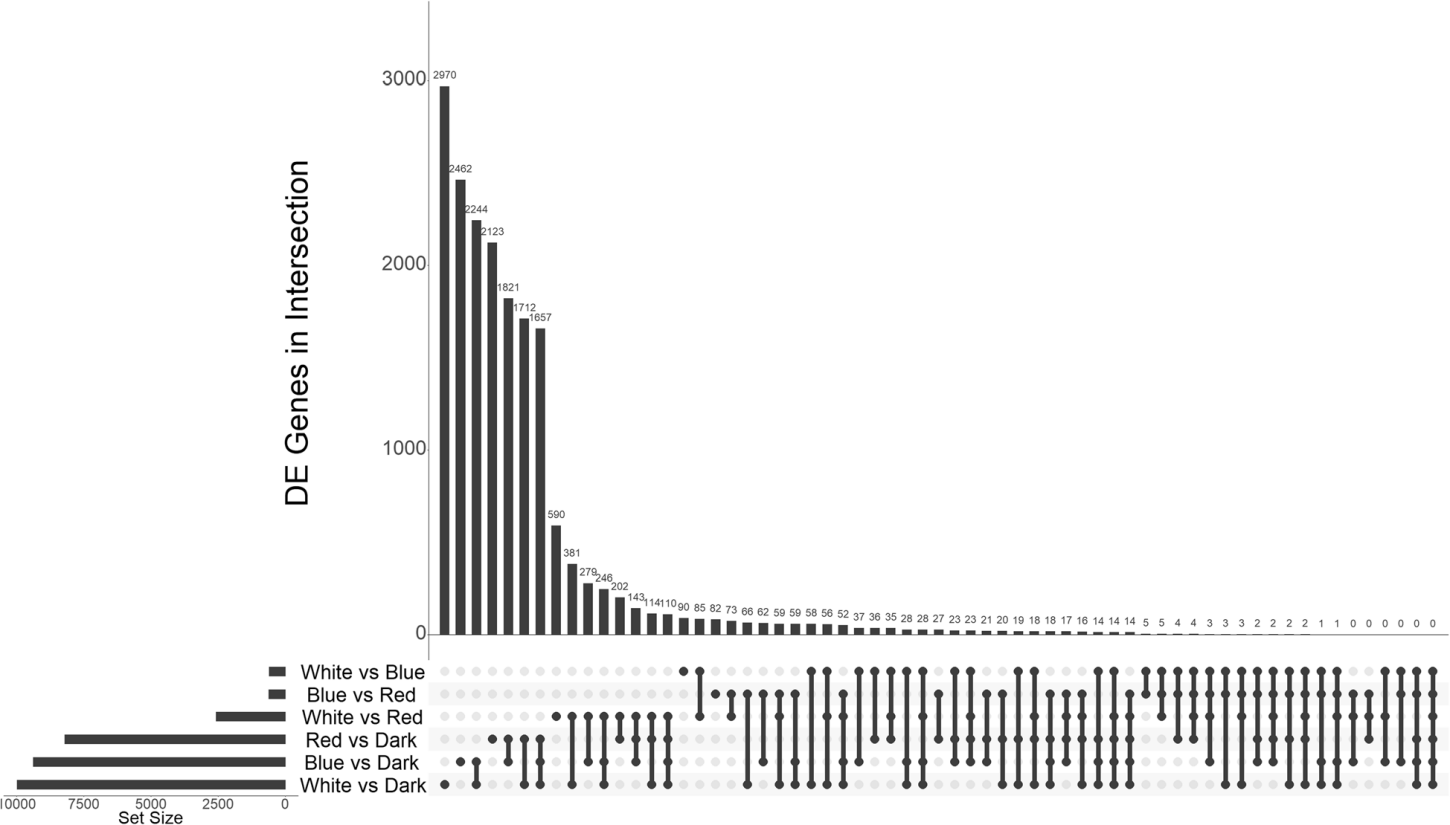
An UpSet plot showing the number of unique and shared differentially expressed genes of *Zymoseptoria tritici* between each comparison [70]

The dark condition had the highest number of unique differentially expressed genes in all three comparisons with the light treatments (Fig. 1). Blue light consistently had the fewest unique differentially expressed genes compared to all of the other treatments (Fig. 1). Many of the genes that are differentially expressed between any light condition and full darkness are differentially expressed between all light conditions and full darkness, with white light having the highest number of unique differentially expressed genes (Fig. 1).

**Fig. 2:**
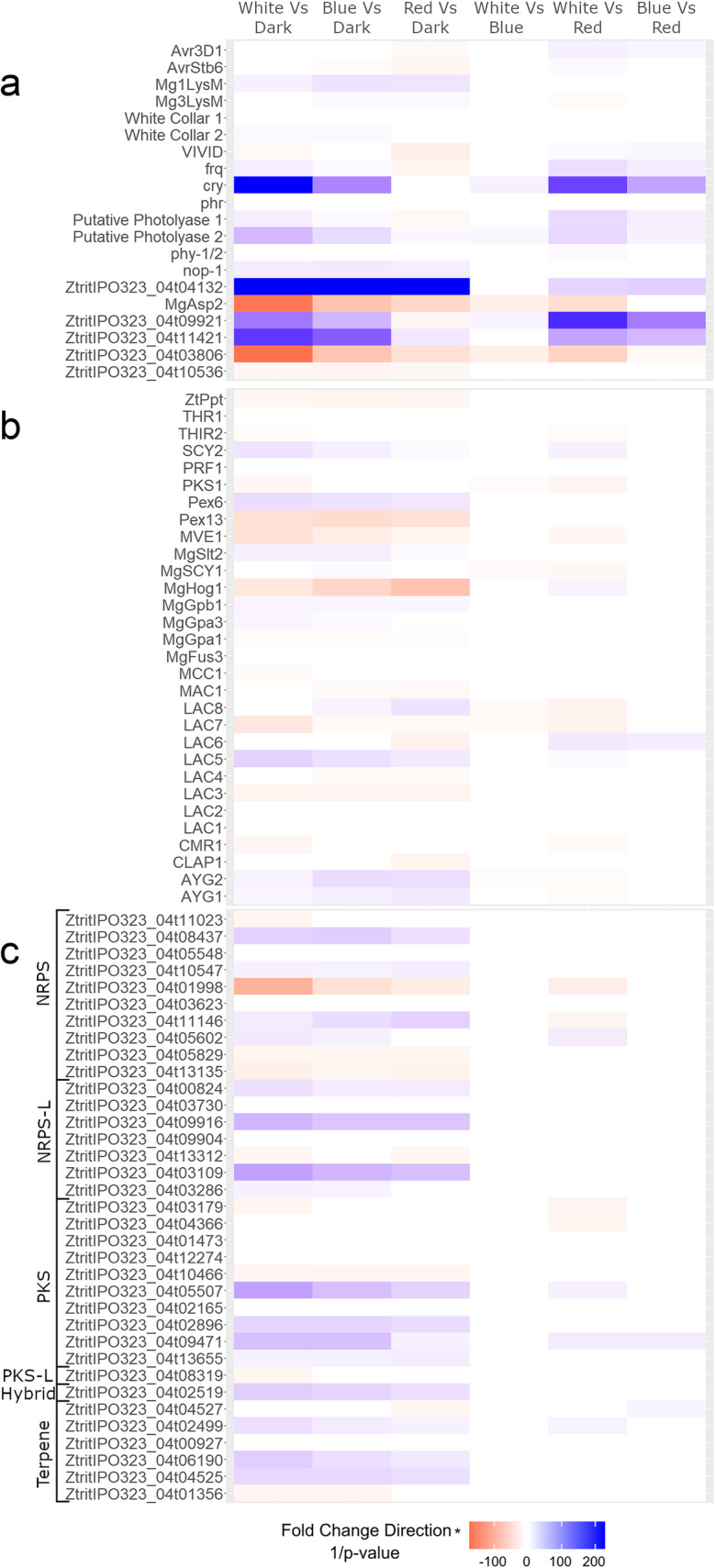
A heatmap of modified adjusted *p* values for selected *Zymoseptoria tritici* genes possibly involved in pathogenicity and light sensing and response. The values are the inverse of the adjusted p value multiplied by the direction of the fold change. **a**. Genes for light sensing, known effector and AVR genes, and selected differentially expressed genes from Additional File 3. **b**. Genes related to melanin production. **c**. Genes related to secondary metabolism genes (NRPS: Non-ribosomal peptide synthetases, NRPS-L: NRPS-like, PKS: Polyketide synthase, PKS-L: PKS-like)

Receptors play an important role in sensing and responding to light. Many of the potential light-sensing proteins in *Z. tritici* were not differentially expressed in any comparison (Fig. 2a). Multiple genes with photolyase-like regions were differentially expressed under various conditions. The cryptochrome/photolyase gene *CRY* was upregulated in white light and blue light, but not in red light or darkness. The rhodopsin-like gene *NOP-1* was upregulated in all types of light versus dark. The known light-signaling gene *MVE1* was upregulated in the dark relative to white light and blue light (Fig. 2b). Interestingly, four genes for effector proteins were differentially expressed under different light conditions, two LysM effectors, Avr3D1, and AvrStb6 (Fig. 2a).

There are three MAPK (mitogen-activated protein kinase)-encoding genes in *Z. tritici* [71–73]. *MgHog1* (ZtritIPO323_04g02798) was strongly downregulated in light, especially in red light (Fig. 2b). Another MAPK-encoding gene, *MgSlt2* (ZtritIPO323_04g00461), was upregulated in light (Fig. 2b). A third MAPK-encoding gene, *MgFus3* (ZtritIPO323_04g10805), was not differentially expressed in any comparison (Fig. 2b). All three of these MAPK-encoding genes are essential for full virulence of *Z. tritici* as well as the production of melanin [71–73], a critical component of photoprotection in this fungus. Another melanization-related gene, *PKS1*, is downregulated in white light versus blue and red light, which indicated that it also may be regulated by a light color such as green, or by an interplay of the blue-light and red-light sensing genes (Fig. 2b) [74].

Three small, secreted proteins (SSP) are highly differentially expressed in various comparisons (Fig. 2a) [75]. ZtritIPO323_04g09921 is strongly upregulated in blue and white light versus red light and dark and is downregulated in red light versus dark. This indicated that expression of this gene might be increased by white and blue light but suppressed by red light relative to dark. Another SSP, ZtritIPO323_04g11421, was highly upregulated in blue and white light versus red light and dark, indicating that its expression may be stimulated by the blue light spectrum. The third SSP, ZtritIPO323_04g03806, is strongly downregulated in all light conditions versus darkness, so may be repressed by all types of light. All three of these SSPs are functionally unannotated, and had no fungal matches in the NCBI NR database. One of the few SSPs with a functional annotation, ZtritIPO323_04g10536, has a non-orthologous group (NOG) of “chitin binding peritrophin-A” domains (10PPR@NOG according to the eggNOG annotation) [76], and is slightly downregulated in light (log2 fold change in white versus dark: − 2.16, blue versus dark: − 2.01, red versus dark: − 1.88).

One protease had a highly significant expression pattern (Fig. 2a) [75]. *MgAsp2* (ZtritIPO323_04g06056) was downregulated in all but one comparison, the exception being blue versus red light. The NOG associated with this gene is 03JP4@ascNOG, a secreted aspartic protease orthologous group, and *blastp* links it to other aspartic proteases [76]. Many other proteases had significant differences in transcript abundance between treatments, with approximately a third of the proteases upregulated, a third downregulated, and a third not differentially expressed in the light versus dark comparisons (Additional File 3D).

No obvious patterns were apparent in differential expression of genes for secreted proteins from other functional classes. Very few secreted lipase genes were differentially expressed in the various light versus dark comparisons (Additional File 3B). Most secreted peroxidases were more differentially expressed in white light rather than blue or red (Additional File 3E). Almost half of the plant cell wall degrading enzymes (PCWDEs) were downregulated by light, but with no consistent pattern (Additional File 3C).

The genes related to secondary metabolism [77] tend to be upregulated by light if they are differentially expressed. Among all the genes, nearly half of them are upregulated, and only a quarter of them are downregulated in light versus dark comparisons (Fig. 2c). In each of the categories of secondary metabolism genes, most had more genes upregulated than downregulated or not differentially expressed (Fig. 2c). The exception is the polyketide synthase-like genes (PKS-L), where there was only one, and it was downregulated only in white light versus dark (Fig. 2c).

Gene ZtritIPO323_04g04132 showed very high differential expression (Fig. 2a). The adjusted *p* value for this gene in the white versus dark and blue versus dark comparisons was 0, with a log2 fold change of 8.7 and 8.9, respectively, and it had an adjusted p value of 1.1e^− 214^ and a log2 fold change of 6.7 in the red versus dark comparison. It was also less dramatically differentially expressed in comparisons between white light versus red and blue versus red. The only functional annotations for this gene are an alpha-beta hydrolase fold (03NR3@ascNOG) and a methyl ester carboxylesterase conserved domain according to the NCBI Conserved Domain Database; it was not annotated as being in a class related to pathogenicity by Palma-Guerrero et al. [75, 76, 78–81].

Among all differentially expressed genes, approximately 3% of those in the light treatments relative to dark were located on dispensable chromosomes that are not present in all isolates of the pathogen [63]; all of the rest occurred on the core chromosome set. Very few genes on dispensable chromosomes were differentially expressed when the three light treatments were compared to each other, ranging from 1 in the blue versus red light comparison to 57 between white light and red (Table 3).

**Table 3:** Numbers of differentially expressed genes on core (numbers 1–13) and dispensable (14–21) chromosomes in the six comparisons of RNA sequences from *Zymoseptoria tritici* cultures exposed to different wavelengths of light or kept in the dark

Chromosome	White versus dark	Blue versus dark	Red versus dark	White versus blue	White versus red	Blue versus red	Total genes
1	548	433	368	6	100	14	2319
2	327	272	233	12	46	2	1393
3	286	235	194	6	67	15	1307
4	244	196	181	7	48	7	998
5	239	196	178	6	43	6	987
6	202	175	144	8	41	5	829
7	189	155	129	9	48	8	822
8	183	156	130	3	26	5	848
9	155	136	110	5	33	2	714
10	148	127	110	2	22	5	616
11	146	129	114	4	24	3	605
12	107	87	77	1	14	3	521
13	95	76	54	3	21	6	423
14	16	13	7	0	3	0	159
15	12	8	9	6	15	1	142
16	12	16	21	2	5	0	164
17	6	3	11	7	15	0	131
18	30	30	25	0	0	0	130
19	13	12	13	1	7	0	139
20	7	3	6	2	9	0	129
21	5	4	9	0	3	0	114
Core 1–13	2869	2373	2022	72	533	81	12,382
Dispensable 14–21	101	89	101	18	57	1	1108
Total	2970	2462	2123	90	590	82	13,490
Percent dispensable	3.4	3.6	4.8	20.0	9.7	1.2	8.2

### Functional characteristics

From 48 to 63% of the genes with significant differential expression in each comparison had no corresponding functional annotation (Table 2, Additional File 2). This includes KOG classes and GO terms in the frozen gene catalog created as part of the original annotation by the Joint Genome Institute [63], as well as EggNOG classifications [76] generated with the analysis of additional RNA sequences by King [82]. On average, 45.5% of genes could be assigned functional protein annotations, while 27.7% could be assigned GO terms. In the full genome, 55.3% of genes have annotations, and 32.8% have GO terms assigned. There is a distinct division between the three light/dark comparisons and the light/light comparisons; the light/light comparisons have a higher percentage of genes annotated to the full genome (average of 59.3%), while the light/dark comparisons have fewer genes annotated than the full genome (average of 49.7%).

The enriched GO terms were quite diverse between the comparisons. The numbers of GO terms enriched between the three light treatments and the dark treatment were higher than those between the light conditions (Table 4). This was similar to the raw numbers of differentially expressed genes (Table 2).

**Table 4:** Number of enriched Gene Ontology terms for the up- and down-regulated genes in each comparison

Direction of change	White versus dark	Blue versus dark	Red versus dark	White versus blue	White versus red	Red versus blue
Up-regulated	138	106	110	1	39	10
Down-regulated	126	177	89	118	148	62

In pairwise comparisons between the light conditions, white light had fewer enriched GO terms than blue or red (Table 4). Compared to blue, white light had only one enriched GO term, protein phosphorylation (GO:0006468), that was also enriched in the dark in all three of the light versus dark comparisons. The gene this GO term was associated with was a serine/threonine kinase. More enriched GO terms were identified when cultures were grown under blue or red light than in the wider-range, white-light condition.

**Fig. 3:**
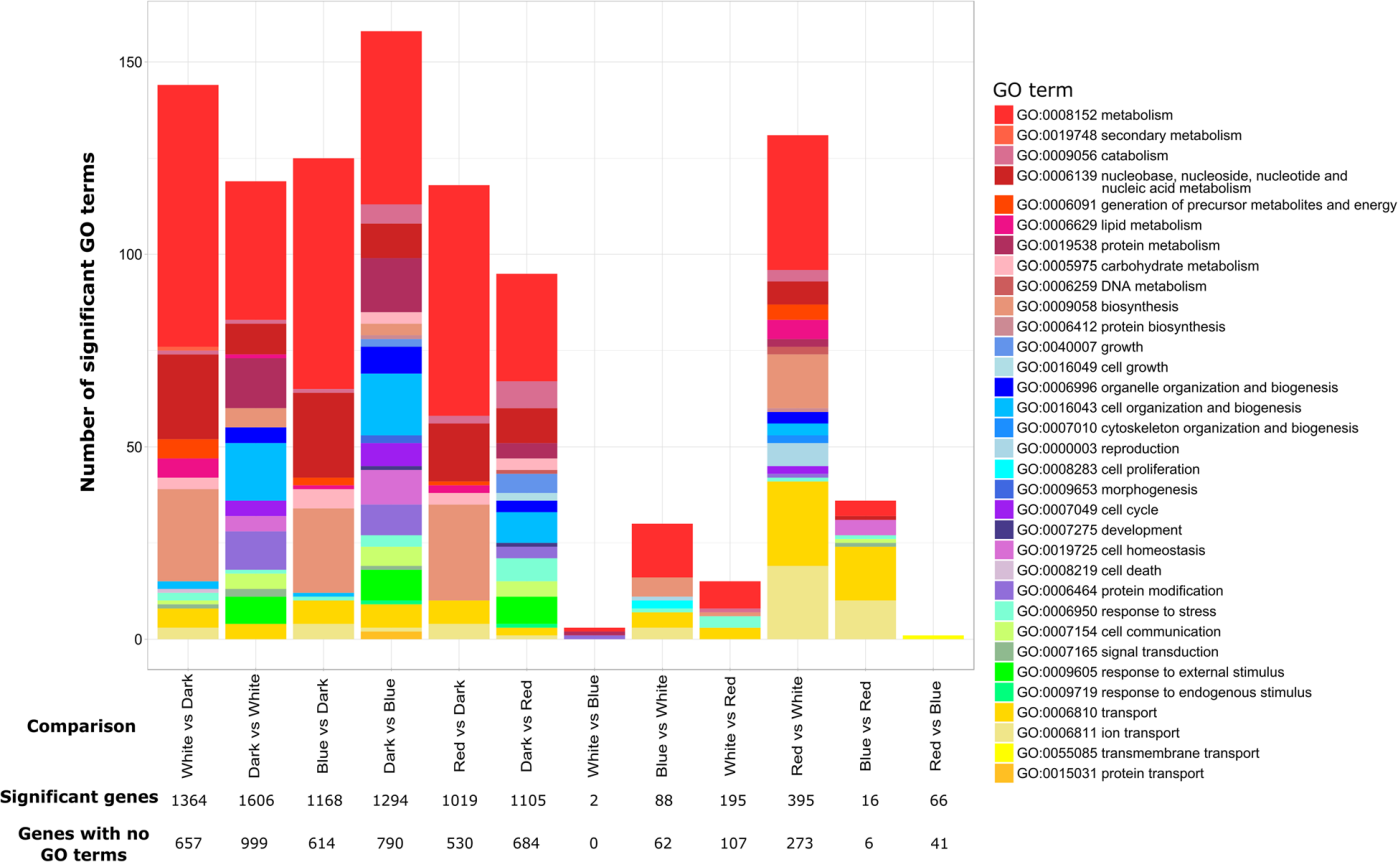
Stacked bar charts showing enriched Gene Ontology (GO) terms in comparisons between *Zymoseptoria tritici* transcriptomes after exposure to different light treatments. The first condition listed is upregulated, e.g., White vs Dark indicates GO terms upregulated in white light versus dark. The first six comparisons from left to right are light treatments compared to the dark, while the remaining six bar charts show comparisons between the different light treatments. Bars show the numbers of significant GO terms in each of the major categories indicated by color according to the legend on the right. Individual genes may have more than one GO term. The total number of differentially expressed genes and the number of those that had no GO terms for each comparison are indicated below the relevant bars

The dark condition had a wider range of enriched GO terms relative to the light conditions (Fig. 3). The variety of enriched GO terms was especially notable in the dark versus blue light comparison (Fig. 3). The dark treatment had more GO terms that were related to growth and development than did cultures grown in the light, where those GO terms occurred rarely. This was very apparent in the blue light and red light versus dark comparisons, where multiple GO terms for growth were enriched in the dark in each comparison. These GO terms included the high-level growth GO term (GO:0040007), as well as filamentous growth (GO:0030447), chromosome segregation (GO:0007059), cellular component biosynthesis (GO:0044085), and numerous cellular components related to mitotic division (GO:0000776, GO:0000794, GO:0000942, GO:0000793), among others.

The light conditions versus dark had many more enriched GO terms related to metabolism (Fig. 3 in red) as well as some that were related to transportation of substances in the cell. The dark versus white and dark versus blue comparisons were enriched in GO terms related to growth while the white versus dark comparison contained several GO terms related to communication.

The comparisons between the three light treatments had fewer enriched GO terms compared to the light versus dark comparisons. The exception to this was the genes that were downregulated in white versus red light, which looked more like a comparison with a dark treatment than did the white versus red and blue versus red comparisons (Fig. 3).

Many of the light comparisons have enriched GO terms for responses to oxidative stress, including those that are enriched on both sides of a comparison, such as the general response to oxidative stress (GO:0006979), which was enriched in both white and red light. White and blue light had more of these GO terms enriched than red light, and dark had none. These include response to oxidative stress (GO:0006979), a response to hydrogen peroxide (GO:0042542), peroxidase activity (GO:0004601), and multiple oxidoreductase-related GO terms (GO:0016684, GO:0016705, GO:0016634, GO:0006733, GO:0016634, GO:0016722).

**Fig. 4:**
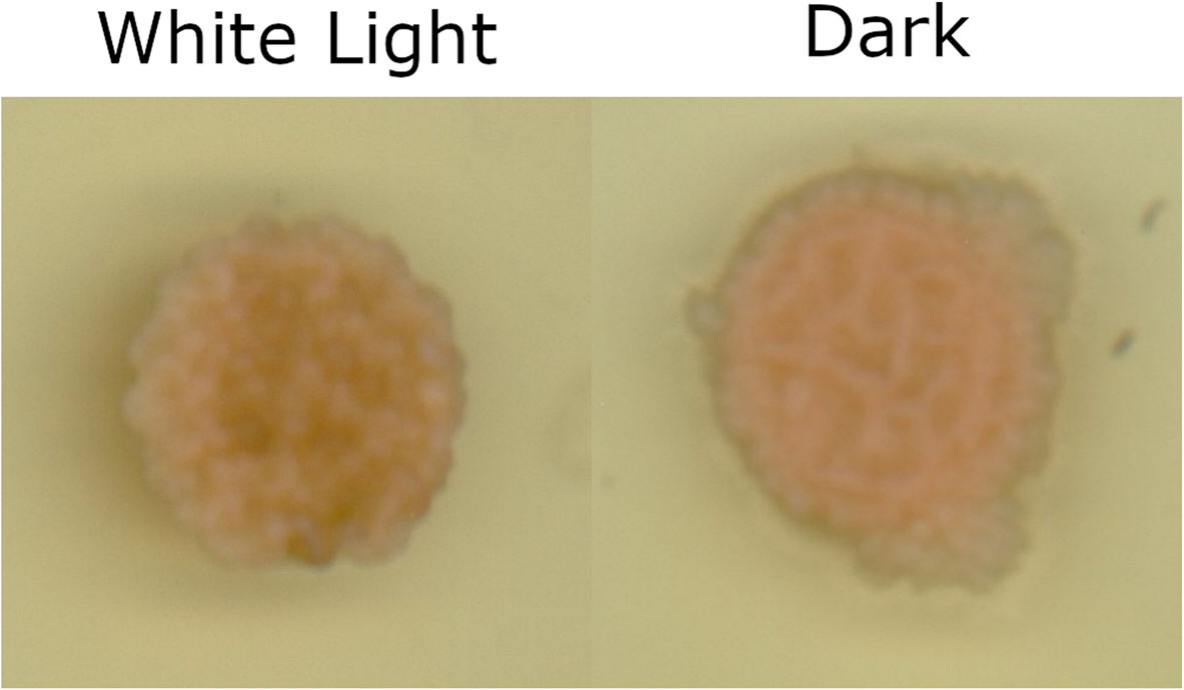
Comparison of *Zymoseptoria tritici* cultures grown in day:night cycles and constant dark on yeast-sucrose agar (YSA) plates. Drops (2 μL) containing approximately 2x10^4^ cells were inoculated on to YSA plates and grown under white light or dark

The KOG functional annotations had similar results to the GO annotations, showing that more genes related to the transport and metabolism of major substances are upregulated in the light rather than the dark. The class “defense mechanisms” also had more genes upregulated in the light than in the dark. The opposite trend occurred with some KOG classes related to growth, which had more genes upregulated in the dark. These KOG classes include “cell cycle control, cell division, chromosome partitioning”, “cell wall/membrane/envelope biogenesis”, “signal transduction mechanisms”, “replication, recombination and repair”, and “intracellular trafficking, secretion, and vesicular transport”. These differences did not appear in the light versus light comparisons. Despite these changes in transcription of genes involved in growth and development and previous studies [57, 58], there were no obvious differences in the morphologies of cultures grown in the dark versus any of the light conditions (Fig. 4).

## Discussion

The RNAseq analyses show clearly that *Z. tritici* can sense and respond to light. The high numbers of differentially expressed genes between the comparisons indicate that *Z. tritici* alters its transcriptome in the dark compared to when it is growing in the light and between different wavelengths of light. While this is not surprising, as many other fungi have been shown to sense and respond to light, this is the first confirmation that the hemibiotrophic wheat pathogen *Z. tritici* also uses light to trigger large differences in gene transcription, which presumably has a correspondingly large effect on metabolic processes [15, 20, 34, 83, 84].

Approximately half of the differentially expressed genes (48–63% depending on the comparison) do not have any functional annotations so this is the first time they have been implicated in possible responses to light or dark. The proportions of unannotated genes among those that were differentially expressed in the light and dark treatments were moderately higher than their representations in the genome in general. This could be in part because no other analyses of differential expression in response to light have been performed on *Z. tritici*, but also could indicate that this fungus has different light responses than those that have been seen in other species. Many prior analyses of light responses in fungi have been with the model fungus *N. crassa* in the class Sordariomycetes. Light responses of fungi in the class Dothideomycetes to which *Z. tritici* belongs are less well understood, and it is possible that this species may use different genes or have different responses compared to other fungi. The differentially expressed genes with no functional annotations could indicate that novel pathways are involved in the responses of Dothideomycetes to light. This hypothesis could be tested in the future by knocking out those genes to identify any altered phenotypes in response to different light conditions.

Light affected expression of genes on all 21 chromosomes in the *Z. tritici* genome, including all eight dispensable (or accessory) chromosomes, indicating that light most likely has a significant effect on growth and development of this fungus. The percentages of genes that were differentially expressed in any of the comparisons agrees with what was found previously in *N. crassa*, indicating that while the response was large, this is likely correct [16]. One interesting result was that genes with annotations relating to oxidative stress, metabolism and transportation were upregulated primarily in the light, while the fungus would be growing on its host during the day, while genes with annotations relating to growth and general cell maintenance were more common primarily in the dark, and therefore would be expressed at night. Further experiments are needed to test whether these in vitro experiments reflect gene expression on the host. Unlike the pattern seen in *Z. tritici*, in *Trichoderma atroviride*, transport-related genes were repressed in the wild type when grown under constant light and were expressed in a *wc1* knockout mutant [85]. However, our findings on cellular metabolism are similar to what was found in *N. crassa* previously [16, 86].

The upregulation of genes related to oxidative stress under light indicates another common way that fungi can respond to radiation-induced stresses. Oxidoreductases are involved in reducing free radicals generated by UV radiation so their increased expression in response to light would be adaptive for the fungus. Light has been shown to increase a response to oxidative stresses in other organisms [15, 16, 20, 85] and seems to induce similar responses in *Z. tritici*.

Expression patterns for many genes were as expected based on their predicted biology. For example, cryptochromes are involved in sensing and responding to blue light in other organisms [10, 87]. The primary cryptochrome gene in *Z. tritici*, *Cry*, is upregulated very specifically in white and blue light as would be expected from its biological function. Photolyases repair DNA damage caused by exposure to light, particularly UV [88]. One of the two putative *Z. tritici* photolyase genes analyzed was strongly upregulated in response to blue and white light, which would be the closest to UV, while the other was upregulated at a low level. The main photolyase gene, *Phr*, was not differentially expressed in response to light so may have a different function or could be expressed constitutively to protect against DNA damage in general.

In contrast, many genes with light-related biological functions in other organisms were not differentially expressed in the *Z. tritici* experiments. This included light-sensing genes such as *wc1* and the phytochrome genes (*Phy-1/2*). While they may not be differentially expressed under long exposure to light, such as that used here, it is possible that other results would be obtained during transitory periods of light, such as during dawn or dusk, which affects *wc-1* in *N. crassa* [16]. Many of these genes may be regulated in a similar fashion, with higher differential expression when light conditions are changing. Another possibility is that the major light-sensing proteins are expressed constitutively so that they are available to detect changes when they occur. Additional experiments covering diurnal variations are required to answer these questions.

Proteins likely to be involved in fungal virulence showed some interesting expression patterns. The most obvious of these were the increased transcription of LysM domain-containing genes in response to light. These code for effector proteins that can minimize wheat host defenses in response to *Z. tritici* during the initial invasion phase of infection by binding chitin, and protecting the cell wall from hydrolysis [89]. *Mg1LysM* was upregulated by any light, while *Mg3LysM* was upregulated primarily by red light. *Mg3LysM* is the effector primarily responsible for blocking the wheat host from activating defenses [89], so it is interesting that it would be upregulated in red light, rather than be expressed constitutively. Similarly, while *Mg1LysM* is not actively responsible for blocking a defense response, it is not clear why it would be expressed in the light rather than constitutively unless it has another role related to growth or pathogenicity.

Some other genes that could be linked to pathogenicity, including proteases and SSPs, also showed differential expression in response to light. Very few of the differentially expressed SSPs had any functional annotations other than being flagged as such by Palma-Guerrero et al. [75]. One of those with an annotation that was downregulated in response to light, ZtritIPO323_04g10536, had a chitin-binding peritrophin-A domain. These domains are found primarily in insects [76], and most of the non-orthologous group annotations were from *Drosophila* species. Preventing the plant from sensing chitin is critical for avoiding PAMP-triggered immunity and other responses against fungal invaders, and is the major effect of the LysM effector proteins secreted by *Z. tritici* during infection. Curiously, the expression patterns of ZtritIPO323_04g10536 were opposite those of the LysM genes, which were induced by light. This could be an indication that this is another chitin-binding effector that works opposite to the LysM effectors.

The proteases were fairly evenly divided between being upregulated by light, downregulated by light, or not differentially expressed. Expression of proteases is consistent with the conclusion of Goodwin et al. [63] that the genome of *Z. tritici* was more similar to those of endophytes rather than other pathogens and that pathogenicity might involve catalysis of proteins rather than carbohydrates during the early stages of infection. However, why most of those genes would have higher expression in response to light is not known. In contrast to most proteases, the one with the highest differential expression, aspartic protease *MgAsp2*, is part of a family of genes that is potentially involved in pathogenicity in *B. cinerea* [90], yet had much higher expression in dark than in light.

Pigmentation is a critical component of photoprotection, or is controlled by circadian rhythms to produce pigments during the day [15, 17, 20, 44, 91, 92]. Despite this tendency, the pigment-related genes in *Z. tritici* varied in whether they were up or down regulated in the light. The three MAPK-encoding genes provide a good example. All three are required for melanization [71–73], yet each has a different expression pattern. *MgFus3* was not differentially expressed, *MgHog1* was downregulated by light, and *MgSlt2* was upregulated by white and blue light (Fig. 2b). These genes are also required for full virulence, via different parts of the infection cycle [71–73]. Another gene that is known to be directly involved in the synthesis of melanin, the polyketide synthase PKS1 [74], was downregulated in white light only. It was not differentially expressed in any other comparison, indicating that it may not be photoregulated or is regulated by a color of light outside of blue and red. We found that melanization of *Z. tritici* hyphae occurs independent of light conditions, and this further supports the hypothesis that melanization is not regulated by light in this fungus*.*

Other genes in the secondary metabolism pathways also are differentially expressed and tend to be upregulated in the light. Nearly half of them were upregulated in the light, and the remaining half were divided between being downregulated and not differentially expressed (Fig. 2c). This correlates with the findings from the enriched GO terms, where metabolism and secondary metabolism GO classes were expressed more in the light than in the dark (Fig. 3).

There are many other classes of genes that have been linked to pathogenicity in other species of fungal pathogens, including the PCWDEs, peroxidases, and lipases. The effect of light on these genes varied within classes. There were some general trends, such as nearly half of the PCWDEs were downregulated in light, but due to a lack of detailed annotations, more granular regulation trends are difficult to ascertain. A better understanding of how these genes are expressed in the host under various light conditions is essential for a complete picture of gene expression during infection.

This experiment provided a single snapshot of gene expression under different light conditions, rather than analyzing changes over time or during the transitions from dark to light and vice versa. As such, some genes that may be differentially expressed only during a short period after light exposure would not be found. Previous RNAseq experiments in *N. crassa* show that there are genes that are differentially expressed, both up and down regulated, during the 15 to 240 min after initial exposure to light that then return to basal dark expression [16]. It is highly likely that additional *Z. tritici* genes would have been differentially expressed if samples had been collected during the first few minutes or hours after the transitions to light or dark. For instance, *white collar 1* in *N. crassa* was highly differentially expressed during the first 15 min following exposure to white light, but returned to dark levels of transcription after an hour [16].

It was not possible to conclude which color of light stimulates the greatest response from *Z. tritici*. The expression patterns under red and blue light were very similar, with only a small percentage of genes differing between these two light-versus-dark comparisons. The white light versus dark comparison had the highest number of differentially expressed genes among the three light treatments, but since blue and red are both components of white light this does not help to identify which color can be sensed most efficiently. The genome sequence of *Z. tritici* [63] appears to have more genes for sensing and responding to blue light, including the WCC, VIVID, cryptochrome, and photolyase genes compared to only a single phytochrome gene for red. Biologically it may make sense for the fungus to have a heightened ability to sense and respond to blue light, as this would most likely co-occur with damaging UV, while red light alone would be present in nature mostly when the light is changing near sunrise or sunset and the amount of UV would be lower.

## Conclusions

These limited initial experiments on gene expression show that *Z*. *tritici* can sense and respond to light, with profound effects on growth, development and metabolism. The large number of functionally unannotated genes involved in light responses indicates a huge gap in our knowledge that must be filled before we can fully understand the effects of light on this fungus. We still know nothing about the minimum intensity of light that can trigger a response, how long light must be present before a response is initiated, or whether the fungus can specifically sense UV. Differential expression of various genes potentially or proven to be involved with infection indicates that light could be important for pathogenicity, and other effectors may show a similar pattern of transcription. A better understanding of the effects of light on pathogenicity and other biological processes of this fungus could provide the basis for development of improved disease management strategies in the future.

## Methods

### Fungal growth and light treatments

The *Zymoseptoria tritici* isolate IPO323 was used for these analyses, as it has been sequenced completely, grows readily in culture and, as an excellent reference genome, has been the subject of much prior research [63]. This isolate was grown on sealed yeast-sucrose agar plates (YSA; 10.0 g/L yeast extract, 10.0 g/L sucrose, 15.0 g/L BactoAgar) under filters to control the wavelengths of light received by each culture. The treatments were white light (no filter), blue (400–530 nm) and red (600–700 nm) light using colored acrylic glass filters (American Acrylics, Skokie, IL) [44]. Full dark was achieved by wrapping the plates in aluminum foil. Light for the cultures was provided by 32-W fluorescent bulbs. Each condition had three biological replicates consisting of one 9-cm Petri dish each. The three light conditions had 16:8 day:night cycles. The cultures were initiated with plugs taken from the margins of an actively growing mycelium and grown for a week at 23 °C.

### RNA extraction and sequencing

Fungal tissue was quickly scraped from the agar, frozen immediately in liquid nitrogen for grinding, and total RNA from the cultures was extracted using a QIAGEN RNeasy Plant Mini Kit (Catalog Number 74903), following the manufacturer’s recommendations. The RNA was then sent to the Purdue Genomics Core Facility (West Lafayette, IN) to be processed and sequenced on an Illumina HiSeq 2500. The data were downloaded to the Halstead Computing Cluster of the Purdue Rosen Center for Advanced Computing for analysis.

### RNAseq and statistical analyses

Trimmomatic (version 0.36) was used to remove leading and trailing bases with a phred score lower than 30, and reads that were shorter than 40 base pairs long after base removal [93]. HISAT2 (version 2.0.5) was used to map the remaining reads to the *Z. tritici* reference genome [94–96]. Samtools (version 1.4) was used to sort the mapped reads, and HTSeq-count (version 0.7.0) was used with the King Rothamsted reference annotation to obtain a counts table for genes [82, 97, 98].

The creation of the reads library was done by using the paired-end reads from each of three biological replicates for each of the four light conditions. The table of gene read counts was exported to R (version 3.4.0). The gene read counts were cleaned by removing any genes that had a rowsum of fewer than 100 reads totaled over all samples, and any genes where any one replicate had fewer than 10 reads, in that order. This conservative approach was used to limit the analysis to genes with reliable data for all replicates. DESeq2 (version 1.15.51) was used to calculate differential gene expression and to obtain adjusted *p* values and log2 fold changes. UpSetR (version 1.4.0) was used to generate the UpSet plot [70].

A custom Perl script was used to pull gene sequences from the reference annotation and the gene sequences were analyzed by eggnog-mapper (version 4.5.1) and InterProScan (version 5.24–63.0) [76, 99]. Another custom Perl script was used to collect each GO term assigned to each gene in a gene-to-GO table, which was fed in to TopGO (version 2.28.0) [100]. These Perl scripts are available on request or from GitHub. Lists of differentially expressed genes from DESeq2 were used to determine overexpressed GO terms found in each comparison.

GOOSE (GO Online SQL Environment) was used to query the Gene Ontology (GO) database to generate levels [101, 102]. The maximum tree depth was used to organize the enriched GO terms for visual analysis. CateGOrizer was used to map the biological processes of each enriched GO term to a parent term in the standard GO_slim [103]. *ggplot2* was used to generate stacked bar charts from the CateGOrizer data [104].

## Supplementary information

**Additional file 1.png** Quality-control checks of the read libraries. A, To the right of the treatment diagonal: Scatterplots comparing two replicates on a log10 scale; To the left of the treatment diagonal: Individual histograms showing the gene read count distribution over each replicate. Treatments of white, blue and red light, and dark are indicated in the diagonal by W, B, R and D, respectively. Replication number is indicated by an integer from 1 to 3, e.g., W2 is the second replication of the white light treatment. B. Histogram of the gene read sums across all replicates. The x axis is the log_10_ read count and the y axis is the number of genes. C. Volcano plots of the log2 fold changes versus the adjusted *p* values.

**Additional file 2. **All genes that were differentially expressed in the six possible comparisons between the dark and three light treatments. Each tab contains the differentially expressed genes in a single comparison, where the first treatment listed is the one showing up regulation, e.g., White versus dark shows all genes that were significantly upregulated under white light compared to the dark at a significance level of *p* = 0.05 or less.

**Additional file 3. **Heatmaps of modified adjusted p values for selected *Zymoseptoria tritici* genes possibly involved in pathogenicity and light sensing and response. The values are the inverse of the adjusted p value multiplied by the direction of the fold change. Comparisons are indicated at the top of each column. The classes A-E are from Palma-Guerrero et al. [75], where: A is small secreted proteins; B are secreted lipases; C shows plant cell wall degrading enzymes; D summarizes protease genes; and E indicates secreted peroxidases.

## Data Availability

The datasets generated and/or analysed during the current study are available in the SRA repository, under accession number SRP151591. Custom Perl scripts are available from the authors upon request, or from GitHub under project name Z.triticiLightRNAseq at home page https://github.com/CBMcCorison/Z.triticiLightRNAseq.

## Abbreviations

WCC: White Collar Complex
wc1: White-collar 1
SDHIs: Succinate dehydrogenase inhibitors
MVE1: *Zymoseptoria tritici* Velvet gene
CRY: Cryptochrome/photolyase gene
NOP-1: Rhodopsin-like gene
Phr: Photolyase gene
Phy-1/2: Phytochrome gene
MAPK: Mitogen-activated protein kinase
SSP: Small secreted protein
NOG: Non-orthologous group
PCWDE: Plant cell wall degrading enzymes
NRPS: Nonribosomal peptide synthetase
PKS: Polyketide synthase
YSA: Yeast-sucrose agar
