# Supplementary figures and images for: The wheat pathogen Zymoseptoria tritici senses and responds to different wavelengths of light
Source: BMC Genomics. 2020 Jul 25;21:513. doi: 10.1186/s12864-020-06899-y (PMC7382159; doi:10.1186/s12864-020-06899-y)

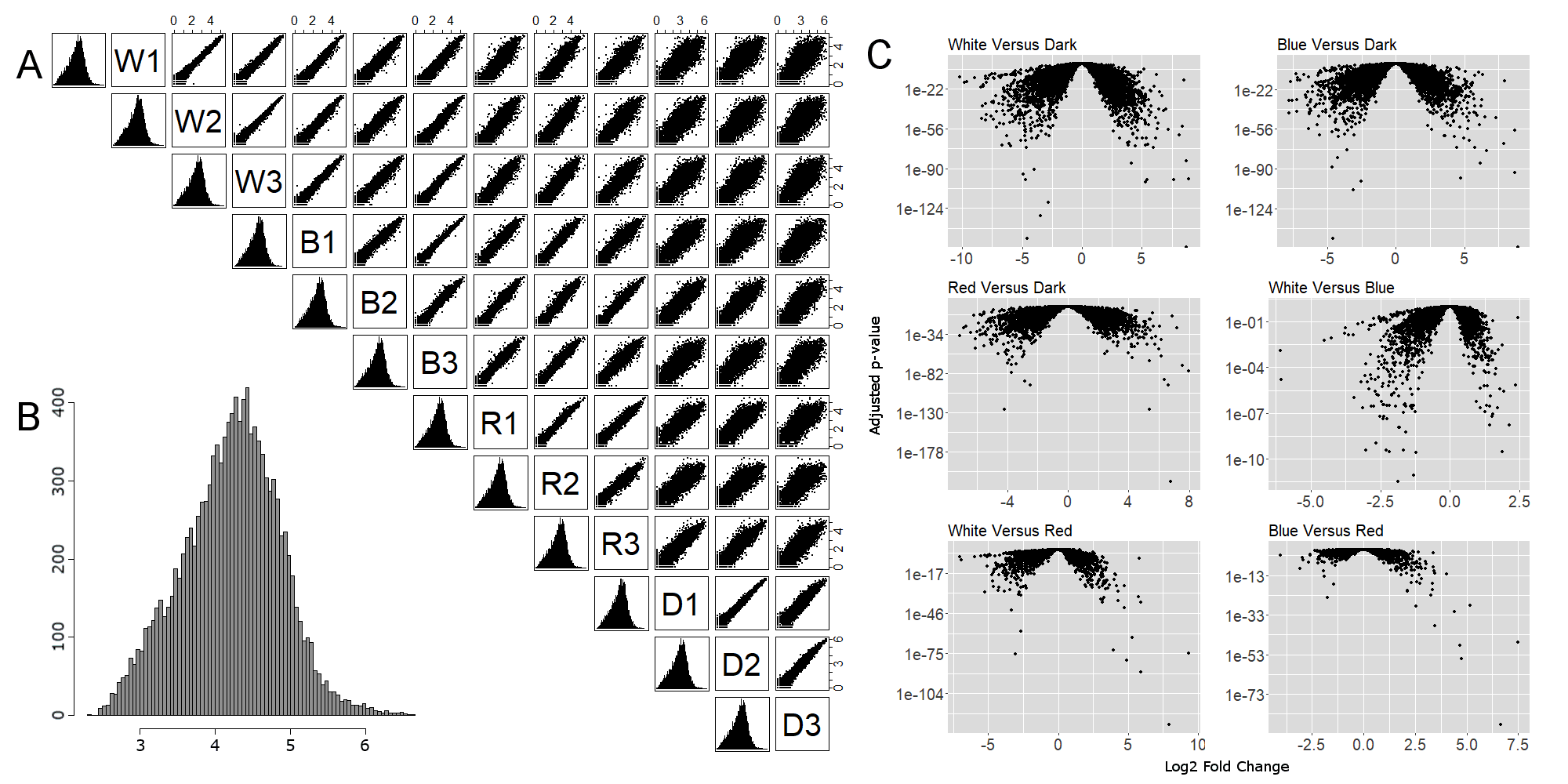

Supplement: Supplementary file 1 — Additional file 1.png Quality-control checks of the read libraries. A, To the right of the treatment diagonal: Scatterplots comparing two replicates on a log10 scale; To the left of the treatment diagonal: Individual histograms showing the gene read count distribution over each replicate. Treatments of white, blue and red light, and dark are indicated in the diagonal by W, B, R and D, respectively. Replication number is indicated by an integer from 1 to 3, e.g., W2 is the second replication of the white light treatment. B. Histogram of the gene read sums across all replicates. The x axis is the log10 read count and the y axis is the number of genes. C. Volcano plots of the log2 fold changes versus the adjusted p values. [file 12864_2020_6899_MOESM1_ESM.png]

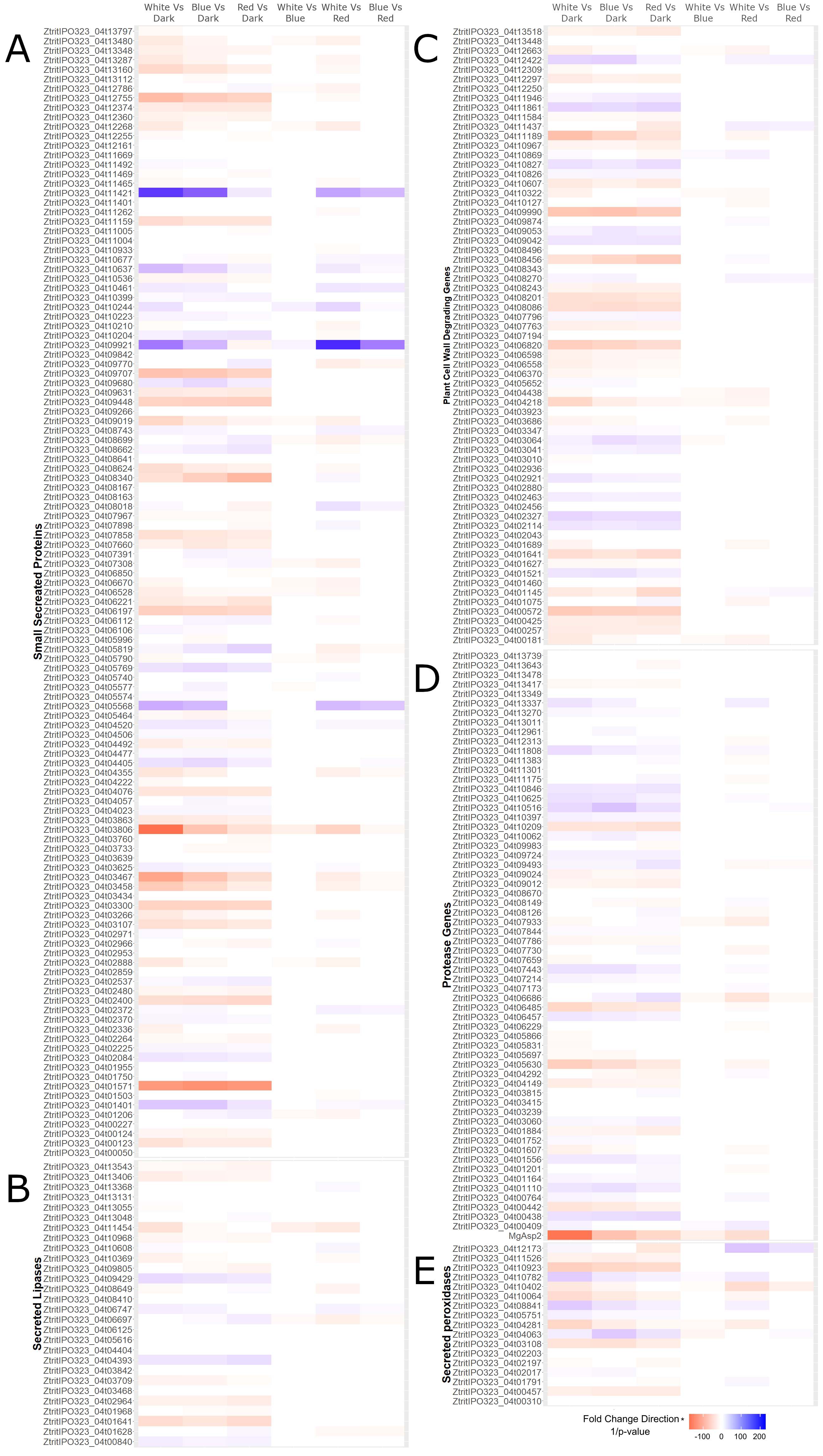

Supplement: Supplementary file 3 — Additional file 3. Heatmaps of modified adjusted p values for selected Zymoseptoria tritici genes possibly involved in pathogenicity and light sensing and response. The values are the inverse of the adjusted p value multiplied by the direction of the fold change. Comparisons are indicated at the top of each column. The classes A-E are from Palma-Guerrero et al. [75], where: A is small secreted proteins; B are secreted lipases; C shows plant cell wall degrading enzymes; D summarizes protease genes; and E indicates secreted peroxidases. [file 12864_2020_6899_MOESM3_ESM.png]
